# Surgical Management of Cerebellopontine Angle (CPA) Lipoma Presenting As Trigeminal Neuralgia: A Case Report

**DOI:** 10.7759/cureus.28082

**Published:** 2022-08-16

**Authors:** Tanaya Dudulwar, Sachin Agrawal, Ramanuj V Kabra

**Affiliations:** 1 Neurological Surgery, Jawaharlal Nehru Medical College, Datta Meghe Institute of Medical Sciences, Wardha, IND; 2 Department of Medicine, Jawaharlal Nehru Medical College, Datta Meghe Institute of Medical Sciences, Wardha, IND; 3 Neurosurgery, Government Medical College and Super Speciality Hospital, Nagpur, IND

**Keywords:** hypoaesthesia, facial nerve palsy, radical surgery, cp angle, pain, hearing loss, trigeminal neuralgia, hamartoma, lipoma, benign tumour

## Abstract

The report details an instance of a 35-year-old male, who came to our hospital with a two-year history of typical trigeminal neuralgia on the left side, predominantly in V2 and V3 dermatomes. The patient was started on medications, but pain could not be controlled by increasing doses and adjuvant medications over the last three months. A lesion was seen on the MRI in the left cerebellopontine angle (CPA) region, hyperintense on T1W and decreased on fat-suppressed imaging, characteristic of lipoma. Being an intractable case, surgery was offered to the patient. Following this, a left retromastoid suboccipital craniectomy was done, and lipoma was excised, decompressing the trigeminal nerve and relieving neuralgia symptoms. In the post-op period, the patient was completely pain-free, but he developed left-sided hearing loss and left facial palsy, Brackmann grade 4, which improved to Brackmann grade 3 on the three-month follow-up.

## Introduction

Tumors of many different forms can develop in the cerebellopontine angle (CPA). The tissue from which the tumors originate determines their distinctive morphological characteristics. As a result, cholesteatomas, osteomas, meningiomas, gliomas, ependymomas, and cysts may develop. The pons, cerebellum, and petrous temporal bone form the boundaries of the CPA cistern, which is filled with cerebrospinal fluid. Cross-sectional photographs make it simple to identify the masses in this area. An algorithmic technique combining morphologic and enhancement properties with well-established demographic data can simplify differential identification of masses in this area. Adipose tissue growth is a characteristic of the benign tumor known as lipoma. It infrequently occurs in the intracranial compartment, accounting for only 0.08% of intracranial tumors, and is found in the interhemispheric fissure in approximately 45% of all intracranial lipomas [1]. In terms of the location of the tumor's origin and surrounding structures, clinical features vary pathologically. Acoustic and non-acoustic tumors are two general categories used to classify CPA tumors. In this study, a rare case is presented of CPA lipoma presenting as trigeminal neuralgia in middle-aged adult male.

## Case presentation

**Figure 1:**
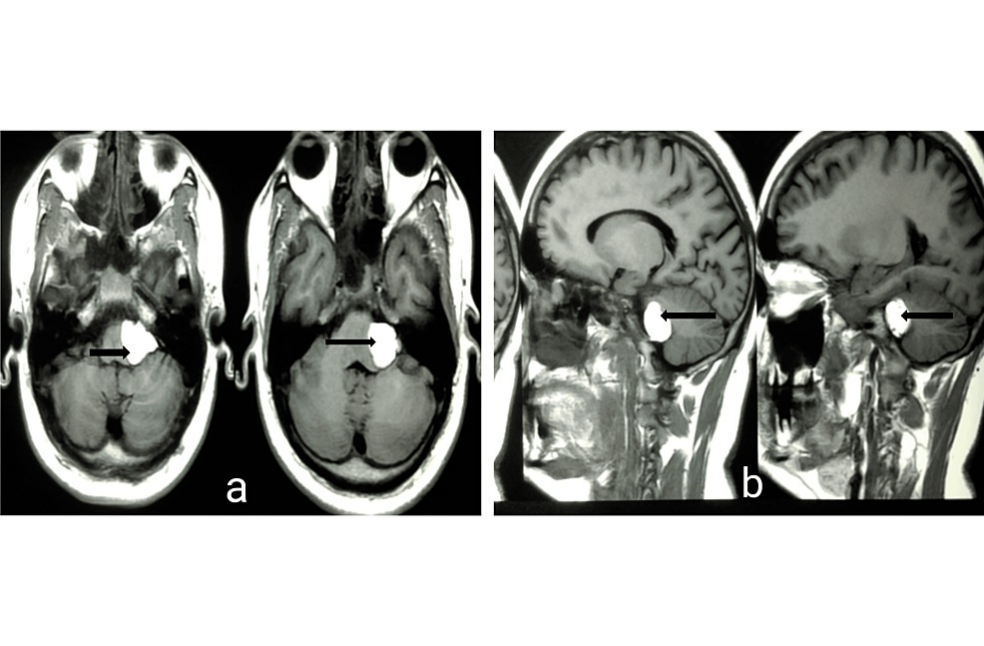
T1W images axial and sagittal section of the brainstem region a) Axial view of brainstem region; b) Sagittal section of brainstem region, denoting an hyperintense   homogenous mass at left cerebellopontine angle (CPA) (arrow). First axial image is higher cut than second axial image, whereas first sagittal image is a lateral cut than second sagittal image, denoting extent of tumor.

**Figure 2:**
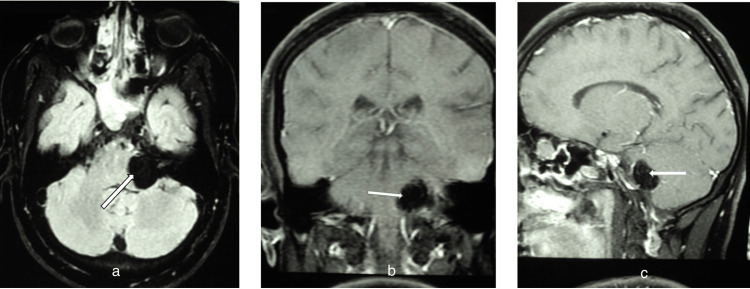
Post-contrast fat saturation sequence MRI a) Axial view showing fat suppression images presenting characteristics of fatty nature of the left cerebellopontine angle (CPA) lesion, which are confirmed in b) coronal and c) sagittal sections.

A middle-aged adult male came with complaints of shooting and burning pain on the left side of the face, predominantly in the mandibular area and in the area of the left cheek for the past two years. The pain used to be intermittent, with episodes lasting for three to five minutes. The pain increased on talking, chewing, and brushing his teeth. The attacks caused great distress to the patient in the sense that he was not able to eat correctly or talk. His oral hygiene also suffered a setback. He got treatments considering dental aetiology in the past but was not relieved. His neurological and ontological examinations were normal, especially the corneal reflexes. Facial sensitivity revealed involvement of V2 and V3 dermatome. Patient was advised an MRI as the pain was prolonged and was not relieved over-the-counter pain relievers. Gadolinium-enhanced MRI brain was done, which revealed an extra-axial, homogenous hyperintense T1W lesion in the left CPA region as shown in Figure 1 (2.3 x 2.5 x2.3 cm), which did not enhance on gadolinium contrast, and the signal from the lesion disappeared on fat saturation sequencing as shown in Figure 2, suggesting the diagnosis to be a lipoma.

**Figure 3:**
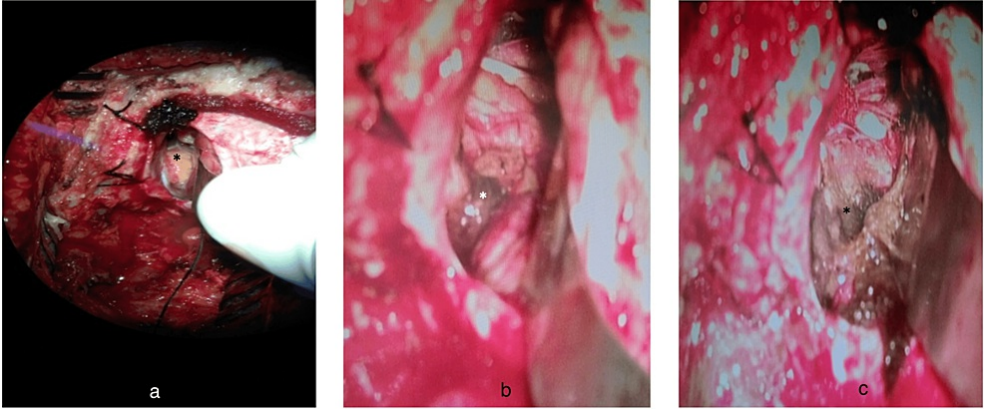
Intra-operative surgical images of cerebellopontine angle (CPA) lesion a) The yellow lesion in the left CPA region (asterisk) engulfing the seven-eighth nerve complex; b) Lipoma abutting the left trigeminal nerve; c) Decompressed left trigeminal nerve with visible vascular loop (asterisk)

The patient was started on tablet carbamazepine 200 mg twice daily. The pain was not controlled on increasing doses and adjuvant medications over the last three months in the form of tablet carbamazepine 300 mg thrice daily, tablet oxcarbamazepine 50 mg twice daily, and tablet pregabalin 75 mg twice daily. Considering it an intractable case, surgery was offered to the patient explaining the risks and complications associated with it and considering the distress related to the lesion, not controlled on medications, and the patient consented to the surgery. In the sitting position, left retromastoid suboccipital craniectomy was done, and a yellowish mass was noted in the left CPA region, engulfing the seven-eighth nerve complex and abutting trigeminal nerve. A vascular loop was found to be compressing the trigeminal nerve because of the mass effect of lipoma, and excision of lipoma resulted in microvascular decompression of the nerve, relieving the symptoms of neuralgia in the post-op period (Figure 3).

 In the post-op period, the patient was completely pain-free, but he developed left-sided hearing loss and left facial palsy, Brackmann grade 4, which improved to Brackmann grade 3 on the three-month followup. 

## Discussion

Intracranial lipomas represent 0.06-0.46% of all intracranial tumors on autopsy, but clinically they represent <0.1 % of the same. Other tumors associated with CPA are listed in Table 1 according to the findings we get on imaging [2].

**Table 1:** Classification of cerebellopontine angle (CPA) masses based on their radiological findings

Site of lesion	Intensity on MRI	Location	Examples	
cerebellopontine angle (CPA)	enhancing	extra-axial		schwannoma, meningioma, metastasis, melanoma, sarcoidosis, tuberculosis, aneurysm
		intra-axial and intraventricular		lymphoma, glioma, metastasis, hemangioblastoma, medulloblastoma, papilloma, ependymoma, dysembryoplastic neuroepithelial tumor
		skull base		paraganglioma, chondromatous tumors, chordoma, endolymphatic sac tumor
	non-enhancing	high T1		lipoma, dermoid cyst, neurenteric cyst, cholesterol granuloma
		low T1		epidermoid cyst, arachnoid cyst, neurocysticercosis

The most common site is pericallosal (>50%), with CPA forming only 9% (60% of CPA lipomas are left-sided) [3]. Lipomas are benign tumors composed of fatty tissues. They may vary in size and number depending on duration and severity. Signs and symptoms seen due to CPA lipomas depend on nerve structures of this region: hearing loss (62%), dizziness (45%), trigeminal neuralgia and sensory impairment in the distribution of the fifth cranial nerve (14%), and facial dysfunction (9%) [4]. Classic trigeminal neuralgia is associated with neurovascular compression in the trigeminal root entry zone, which can lead to demyelination and a dysregulation of voltage-gated sodium channel expression in the membrane. These alterations may be responsible for pain attacks in trigeminal neuralgia patients. It can be managed by giving anticonvulsants (usually carbamazepine) or antispasmodic drugs such as baclofen or botox injection. If there is no improvement, then dosage is increased. Surgical interventions include microvascular decompression and brain stereotactic radiosurgery. Pathologically, they are neither hamartomas nor true neoplasms, just malformation that develops from the continuation of primitive meninx, the predecessor of pia mater and arachnoid mater, that extends into fat. Lipoma at CPA has shown to extend into surrounding structures, i.e., nerve bundles and fasicles, in many previous reports. It compresses nearby nerves, due to which patient present with neurological symptoms, and even after excision of lipoma, can have neurological deficits [5]. MRI brain helps diagnose CPA lipomas, which appear T1W homogenously hyperintense with no gadolinium uptake and no intensity on fat suppression, which rules out other possible differential diagnoses [6]. 

The radical surgery resulted in neurological deficits found to be minimum in the non-radical group [7]. Thus, limited surgery in partial excision should be considered if symptoms are not controlled on medications to avoid permanent complications. Our case was that of a CPA lipoma with intractable pain not controlled on medications even though non-radical excision surgery resulted in neurological deficits in the form of hearing loss and grade 4 facial palsy.

## Conclusions

Males are more likely to develop CPA lipomas, an uncommon benign pathology. Medical management should be attempted to relieve the patient's symptoms, but if it is intractable, then surgery should be recommended. It is strongly advised to use conservative nonoperative care due to poor surgical results and the unusual growth of lipomas. CPA lipomas should only be surgically removed if the symptoms are unbearable, progressively worsen, or the tumor is growing. Limited surgery in partial excision should be considered if symptoms are not controlled on medications to avoid permanent complications like hearing loss, facial nerve palsy, hypoaesthesia, cerebellar signs, or even no improvement. Lipoma has been shown to infiltrate the nerve bundles and be present between the fascicles, so excision of lipoma commonly results in neurological deficits.
